# The Effects of a Nutrition Education Intervention on Sports Nutrition Knowledge during a Competitive Season in Highly Trained Adolescent Swimmers

**DOI:** 10.3390/nu13082713

**Published:** 2021-08-06

**Authors:** Wee Lun Foo, Mark A. Faghy, Andy Sparks, Josh W. Newbury, Lewis A. Gough

**Affiliations:** 1 Research Centre for Life and Sport Science (CLaSS), School of Health Sciences, Birmingham City University, Birmingham B9 3TN, UK; Wee.Foo@mail.bcu.ac.uk (W.L.F.); Josh.Newbury@mail.bcu.ac.uk (J.W.N.); lewis.gough@bcu.ac.uk (L.A.G.); 2 Human Sciences Research Centre, University of Derby, Derby DE1 1GB, UK; 3 Department of Physical Therapy, College of Applied Sciences, University of Illinois at Chicago, Chicago, IL 60007, USA; 4 Sports Nutrition and Performance Research Group, Department of Sport and Physical Activity, Edge Hill University, Ormskirk L39 4QP, UK; sparksa@edgehill.ac.uk

**Keywords:** education, swimming, adolescent athletes, knowledge, performance

## Abstract

The aim of this study was to evaluate the effects of a seven-week nutrition education intervention on the sports nutrition knowledge (SNK) of highly trained UK adolescent swimmers. Fifteen national and international adolescent swimmers (males = 5; females = 10, 15.5 ± 1.1 years, 170.2 ± 7.5 cm, 60.3 ± 5.7 kg) participated in the study during seven consecutive weeks of the competitive swimming season. The participants received 30 min of nutrition education once per week in a classroom-based setting after they had completed their regular swim training. An undergraduate sports nutrition student delivered all nutrition education sessions and SNK questionnaires were administered to the participants pre- and post-intervention. The mean total SNK score improved by 8.3% (SD = 8.4%, 95% CI = 4.1–12.6; *p* = 0.006; ES = 1.0) following the nutrition education sessions. On an individual basis, ten swimmers significantly improved their total SNK score, whereas four swimmers did not improve, and one swimmer performed significantly worse after the intervention. Moreover, the swimmers’ knowledge of hydration improved by 22.2% (SD = 20.6%, 95% CI = 11.8–32.6, *p* = 0.004, ES = 1.1) over the seven-week timeframe, which was the only nutrition topic to have a significantly increased knowledge score. The current study therefore suggests that a nutrition education intervention can positively influence the SNK of highly trained adolescent swimmers.

## 1. Introduction

Adolescence is defined as the period between 10 to 19 years of age and is a life phase where future patterns of adult health are often established [1], including the development of dietary habits and lifelong relationships with food [2]. It is also during this time that sporting commitments can dramatically increase, with some young athletes becoming capable of competing at a high level of participation. Adolescent athletes are therefore presented with a unique nutritional challenge since optimal dietary practices are critical to maintain growth, athletic performance, and health to support possible future careers in sport [2].

Swimmers often undertake high training volumes (1–3 sessions per day) at a very young age to facilitate the development of biomechanical technique, physiological capacity, and race skills, all of which contribute to their ability to compete at an advanced level [3]. Combined with the nutritional requirements to support growth and development, this high level of training places a considerably high energy demand on adolescent swimming competitors [3]. This includes greater quantities of macronutrients and micronutrients, such as carbohydrate [4], protein [5], vitamin B1 [6], and zinc [7]. However, previous research has shown that the nutritional practices of adolescent swimmers are less than desirable, with insufficient energy, carbohydrate, calcium, iron, magnesium, and iodine often being consumed [8,9]. These suboptimal dietary practices could have long-term negative implications on swimmers’ health and performance. For instance, chronic low energy availability caused by insufficient energy intake resulted in ovarian suppression among female swimmers and subsequently led to decrements in swimming performance compared to healthy swimmers [10]. Swimmers were also less capable of tolerating a high training load and experienced more muscle fatigue during an intensified training period as a result of insufficient carbohydrate intake [4]. Based on the available literature highlighting nutritional deficiencies in swimming cohorts, it is plausible to suggest further research on interventions to improve the athletes’ dietary practice could be advantageous to health and exercise performance.

One strategy to improve the dietary practices of adolescent athletes is to enhance their sports nutritional knowledge (SNK), which is suggested to be a key determinant of athletes’ food choices [11]. Indeed, previous research has reported that a higher level of SNK correlates with positive dietary habits among athletic populations [12]. Despite this, the SNK of adolescent athletes is consistently shown to be poor, particularly within swimming cohorts [13,14,15]. The poor SNK of swimmers could be related to their sources of nutrition information since only 3% of female collegiate swimmers obtained their nutrition information from a dietitian, compared to the majority that sought advice from parents (12%), coaches (11%), and magazines (10%) [14]. Furthermore, swimmers who had previously attended nutrition classes were found to have a greater SNK than those who had not previously received any nutrition education [15]. These findings suggest that a nutrition education intervention may help to address current gaps within the SNK of adolescent swimmers.

To date, little is known about the impacts of nutrition education on the SNK of adolescent swimmers despite there being well-established benefits on the SNK of adolescents from other sporting backgrounds [16,17,18,19,20]. In the only published study to date, 37 competitive adolescent swimmers from Cyprus improved their SNK (*p* = 0.034) and adherence to a Mediterranean diet (*p* < 0.01) after a half-day nutrition workshop and a supermarket tour [21]. A caveat to these findings, however, was that the SNK questionnaire used in this study had not been validated. Equally, it is plausible that these findings represent only a distinct sociodemographic, and therefore, further research is warranted to explore other populations. Hence, the aim of this study was to evaluate the effects of a seven-week nutrition education intervention on the SNK of highly trained adolescent swimmers from the UK using a validated SNK questionnaire [22]. It was hypothesised that nutrition education would lead to improvements in SNK of highly trained adolescent swimmers.

## 2. Methods

### 2.1. Experimental Design

A quasi-experimental study design was used in this study with cross-sectional data collection before and after the intervention. This study was approved by Birmingham City University Ethics Committee (Newbury/7596/R(B)HELS FAEC) and all participants provided informed consent to be included in the study. The study was conducted in accordance with the Declaration of Helsinki (2013).

### 2.2. Participants

A total of 15 national and international adolescent swimmers (males = 5; females = 10; mean FINA points = 702 ± 55, range 616–801) from a UK-based high performance amateur swimming club took part in this study. Participants’ mean age, height, and weight were 15.5 years (*SD* = 1.1, range 14.0–17.0), 170.2 cm (*SD* = 7.5, range 153.7–180.1), and 60.3 kg (*SD* = 5.7, range 48.4–68.6), respectively. The swimmers’ main competitive strokes include front crawl (*n* = 6), butterfly (*n* = 4), breaststroke (*n* = 4), and backstroke (*n* = 1).

### 2.3. Description of Nutrition Education

Swimmers attended seven nutritional education sessions focusing on different sports nutrition topics (Table 1) that were delivered by an undergraduate sports nutrition student from Birmingham City University. The education was delivered in 30 min sessions in a classroom-based setting once per week after participants had completed their regular swimming practice. The nutrition education curriculum was a modification of the curriculum from the WAVE project [23]. Education was presented via a PowerPoint presentation with the opportunity for discussion and questions following the presentation. Meal planning activities were included during sessions 1, 2, 3, 4, and 6, in which the participants were required to plan their own meals to achieve the recommended carbohydrate and protein requirements on training and competition days.

### 2.4. Questionnaire

The nutrition knowledge section from a previous sports nutrition questionnaire that was validated in high school rugby players [22] was used to assess SNK in the present study. An SNK score was calculated for each swimmer by adding the total number of correct answers from four nutrition topics (energy and refueling, hydration, supplements, and protein). The minimum score that could be obtained was 0 (0%) and the maximum score was 16 (100%). The questionnaire was administrated via Google Forms software (Google LLC, Mountain View, CA, USA) at two time points: (a) the week before the education intervention (pre-intervention), and (b) one week after the education intervention (post-intervention). A link to the online questionnaire was delivered to the participants via a mobile phone instant messaging application (WhatsApp, Inc., Santa Clara, CA, USA).

### 2.5. Statistical Analysis

Descriptive and statistical analyses were undertaken using SPSS for Windows (version 25; IBM, Armonk, New York, NY, USA). Normality of all data was verified by using visual inspection of Q–Q plot, histogram, and Shapiro–Wilk statistics. Paired samples *t*-tests and Wilcoxon signed-rank tests were used (depending on the normality of the distribution of the data variables) to explore the differences in pre- and post-intervention total SNK and score in each topic (energy and refueling, hydration, supplement, protein) with significance set at *p* < 0.05. Effect sizes were calculated as the change score divided by the SD of the change score [24] and were interpreted as trivial (<0.2), small (0.2–0.4), medium (0.5–0.7) large (≥0.8) [25]. The confidence interval of 95% (95% CI) was calculated and the data variables were interpreted as statistically significant if the CI did not overlap zero [26].

## 3. Results

Sports Nutrition Knowledge

**Figure 1:**
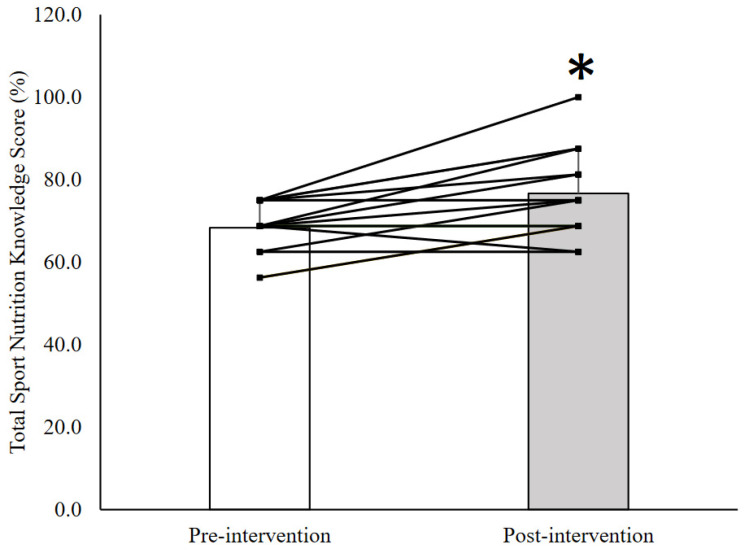
The change in sports nutrition knowledge (SNK) from pre- to post-intervention in highly trained adolescent swimmers. * = a statistically significant change (*p* < 0.05).

The SNK scores for before and after the nutrition education intervention are presented in Table 2. Total SNK score improved by 8.3% (SD = 8.4%; *p* = 0.006; ES = 1.0) following the nutrition education sessions. Out of the 15 participants, an improvement in total SNK score was identified in 10 swimmers, whereas four swimmers showed no change, and one swimmer had a reduced total SNK score (Figure 1). In specific topics, there was a 22.2% (SD = 20.6%, *p* = 0.004) improvement in hydration knowledge, which was further supported by a large effect size for this change (ES = 1.1). A moderate effect size was also calculated for the 13.3% (SD = 27.6%, ES = 0.5) increase in protein knowledge, however, this change did not reach statistical significance (*p* = 0.082). No changes were evident between the pre- and post-intervention scores for energy and refueling or supplement knowledge.

## 4. Discussion

The aim of this study was to evaluate the effectiveness of a classroom-based nutrition education intervention on the nutrition knowledge of highly trained adolescent swimmers. In agreement with our hypothesis, our findings demonstrated that seven weekly nutrition education sessions significantly improved the total SNK of highly trained adolescent swimmers, especially on the topic of hydration. This finding suggests that group-based nutrition education can be an effective tool to enhance swimmers’ nutrition knowledge in a club environment.

The nutrition education intervention in the present study resulted in an 8.3% improvement in the SNK of highly trained adolescent swimmers. This finding corroborates previous research that demonstrated an improvement in nutrition knowledge after a classroom-based group nutrition education intervention among adolescent athletes from various sports, including dancing [18], endurance sport [19], swimming [21], and soccer [20]. Indeed, Patton-Lopez et al. [20] assessed athletes’ SNK using the same sports nutrition questionnaire [22] adopted in the present study and reported a similar magnitude of improvement in SNK (9.3%) among high school soccer players after the two-year intervention that included seven 30 min sports-nutrition educational sessions and three life skills sessions. Furthermore, other studies also reported improvements in SNK; however, direct comparisons were difficult to make with the present findings due to the differences in the SNK questionnaire used [16,17,18,19]. Nonetheless, practitioners can employ a similar nutrition education intervention as used in the current study since this method improved SNK in the majority of participants.

The current nutrition education intervention included seven education sessions with a total contact time of 210 min in a group of 15 participants. It was premature to claim that the intervention used in the present study was the most effective education strategy as previous studies have also improved SNK with contact times ranging from 90 min [16] to 390–490 min [20], with the total number of sessions also varying from one [21] to ten [20]. However, the present study does show that the SNK of an adolescent swimming cohort can be enhanced within a short timeframe. This is important based on the high training volumes that swimmers complete, which alongside school education will often leave them with limited time to focus on their nutrition knowledge and dietary practices. Moreover, this study also suggests that individuals with an educational background in sports nutrition are viable candidates to deliver educational workshops to adolescent athletes. As many other studies employ a qualified dietitian, this offers an alternative strategy for clubs with limited resources. Nonetheless, this study only shows the impact of short-term nutrition educational sessions, and therefore, further studies are warranted to determine the contact time and the total number of sessions required to achieve long-term improvement in SNK.

The number of participants that took part in a session may also influence the outcome of the nutrition education intervention. For example, Reading et al. [27] reported a high dropout rate (81.1%) when the nutrition education session was administered to a group of 30–40 adolescent and young adult male field hockey players at a time. Furthermore, the meal planning activities included in the current study might have played an integral role in enhancing participants’ SNK. These activities likely helped to expand the swimmers’ feelings of autonomy, competence, and relatedness and subsequently translated into a greater intrinsic motivation to gain more knowledge in sports nutrition [28]. Alternatively, it might have been the use of technology that increased the adherence to the program, as previous research has shown that an adherence rate above 80% is possible with the inclusion of mobile phone applications [19]. As this would have not been an option for Reading et al. [27], this might explain why these authors observed such a high dropout rate. Further research investigating the impact that mobile applications can have in increasing interaction with SNK education strategies within adolescents are therefore required.

One of the key limitations of the pre- and post-study design was that there was not a control group; therefore, it is difficult to confidently establish the cause-and-effect relationship between participation in nutrition education sessions and the subsequent improvement in SNK. Furthermore, this study is limited by its sample size (*n* = 15); however, a similar number of participants have been used in previous investigations demonstrating improvements in SNK (*n* = 10–15) [17,29]. In addition, swimmers’ SNK may differ depending on their gender, swimming strokes, and swimming distances, yet these could not be differentiated in the current study due to the limited number of participants from each category. Additionally, relationships between SNK and dietary behaviour (i.e., intake) were not assessed in the present study, and hence, it remains unknown whether the improvements in SNK actually translated into positive changes in sports nutrition practices in adolescent swimmers. Future research should therefore consider including nutritional recalls pre- and post-intervention to provide some insight into the effects that education interventions, such as that in the present study, have on the dietary practices of adolescent athletes.

In conclusion, the results of this study suggest that a seven-week classroom-based nutrition education intervention can positively influence SNK of highly trained adolescent swimmers. The nutrition education intervention used in the present study may serve to provide a framework for sports nutrition practitioners for future application with adolescent swimmers, particularly for the purposes of improving SNK. More research is warranted to explore other interactive ways to deliver nutrition education intervention including supermarket tours, cooking classes, and mobile phone applications or social media-based interventions.

## Figures and Tables

**Table 1:** Nutrition education intervention outline.

Session	Topics	Session Goals
1	Dietary protein and body composition	To understand the importance of dietary protein in optimising body composition and develop the skills necessary to achieve the total daily protein required to facilitate optimal body composition changes.
2	Pre-training nutrition	To understand the purpose of fueling prior to exercise and develop the skills necessary to implement a pre-exercise fueling plan to delay onset of fatigue and dehydration, improve and maintain training and performance, and avoid gastrointestinal discomfort.
3	Nutrition during training and hydration	To understand the purpose of fueling during exercise and develop the skills necessary to implement an intra-exercise fueling plan to improve and maintain training and performance. To understand the purpose of hydration for sport and exercise and develop the skills necessary to implement a hydration plan to delay onset of dehydration, improve and maintain training and performance, and decrease risk of illness and injury.
4	Post-training nutrition	To understand the purpose of recovery nutrition and develop the skills to implement a recovery nutrition plan, including glycogen repletion, rehydration, and initiating recovery and adaptation processes in the body using carbohydrate, protein, and fluids.
5	Nutritional supplements for performance and health	To understand the regulation and safety of nutritional supplements and introduce nutritional supplements with proven ergogenic and health benefits.
6	Competition nutrition	To understand the nutritional requirements during multiple-day swimming events and develop the skills to implement a nutritional plan that improves and maintains performance, including adequate pre-race fueling, glycogen repletion, rehydration, and informed food selection whilst eating at restaurants.
7	Nutrition during taper	To understand the nutritional requirements when training loads are reduced and develop the skills needed to implement a nutrition plan that prevents over- or under-fueling.

**Table 2:** Sports nutrition knowledge (SNK) total score, topic scores, and change scores pre- and post-educational sessions.

	Mean Score				
	Pre-Intervention (%)	Post-Intervention (%)	Pre–Post Changes (%)	SD & 95%CI	*p*-Value & ES
*n* = 15					
Total SNK score	68.3	76.7	8.3	8.4 (95% CI = 4.1–12.6)	*p* = 0.006, ES = 1.0
Energy and refueling	81.0	83.8	2.9	16.4 (95% CI = −5.4–11.1)	*p* = 0.435, ES = 0.2
Hydration	68.9	91.1	22.2	20.6 (95% CI = 11.8–32.6)	*p* = 0.004, ES = 1.1
Supplements	66.7	68.9	2.2	23.5 (95% CI = −9.6–29.5)	*p* = 0.862, ES = 0.1
Protein	40.0	53.3	13.3	27.6 (95% CI = −0.6–27.3)	*p* =0.082, ES = 0.5

## Data Availability

Data sets can be obtained through contacting the corresponding author.
